# Blood–brain and blood–cerebrospinal fluid barrier permeability in spontaneously hypertensive rats

**DOI:** 10.1186/s12987-018-0112-7

**Published:** 2018-09-24

**Authors:** Daphne M. P. Naessens, Judith de Vos, Ed VanBavel, Erik N. T. P. Bakker

**Affiliations:** Department of Biomedical Engineering and Physics, Academic Medical Center, University of Amsterdam, Meibergdreef 9, 1105 AZ Amsterdam, The Netherlands

**Keywords:** Blood–brain barrier, Blood–cerebrospinal fluid barrier, Cerebrospinal fluid, Hypertension, Interstitial fluid

## Abstract

**Background:**

Hypertension is an important risk factor for cerebrovascular disease, including stroke and dementia. Both in humans and animal models of hypertension, neuropathological features such as brain atrophy and oedema have been reported. We hypothesised that cerebrovascular damage resulting from chronic hypertension would manifest itself in a more permeable blood–brain barrier and blood–cerebrospinal fluid barrier. In addition, more leaky barriers could potentially contribute to an enhanced interstitial fluid and cerebrospinal fluid formation, which could, in turn, lead to an elevated intracranial pressure.

**Methods:**

To study this, we monitored intracranial pressure and estimated the cerebrospinal fluid production rate in spontaneously hypertensive (SHR) and normotensive rats (Wistar Kyoto, WKY) at 10 months of age. Blood–brain barrier and blood–cerebrospinal fluid barrier integrity was determined by measuring the leakage of fluorescein from the circulation into the brain and cerebrospinal fluid compartment. Prior to sacrifice, a fluorescently labelled lectin was injected into the bloodstream to visualise the vasculature and subsequently study a number of specific vascular characteristics in six different brain regions.

**Results:**

Blood and brain fluorescein levels were not different between the two strains. However, cerebrospinal fluid fluorescein levels were significantly lower in SHR. This could not be explained by a difference in cerebrospinal fluid turnover, as cerebrospinal fluid production rates were similar in SHR and WKY, but may relate to a larger ventricular volume in the hypertensive strain. Also, intracranial pressure was not different between SHR and WKY. Morphometric analysis of capillary volume fraction, number of branches, capillary diameter, and total length did not reveal differences between SHR and WKY.

**Conclusion:**

In conclusion, we found no evidence for blood–brain barrier or blood–cerebrospinal fluid barrier leakage to a small solute, fluorescein, in rats with established hypertension.

**Electronic supplementary material:**

The online version of this article (10.1186/s12987-018-0112-7) contains supplementary material, which is available to authorized users.

## Background

Chronic hypertension is a well-established risk factor for cerebrovascular disease, including haemorrhagic stroke, vascular dementia and Alzheimer’s disease. It is associated with alterations in the structure and function of cerebral blood vessels, which may contribute to hypoperfusion, microinfarcts, brain atrophy, oedema, and ultimately, cognitive impairment [1, 2]. Although these neuropathological features have been described in many studies, the specific harmful effects of chronic hypertension on these abnormalities remain obscure.

Dysfunction at the blood–brain barrier (BBB) is often regarded as an early and common denominator in cerebrovascular disease. Hypertension could be envisioned to enhance loss of BBB integrity, leading to increased solute permeability of this barrier and leakage of water into the brain parenchyma [1, 2]. The brain extracellular fluids consist of cerebrospinal fluid (CSF), interstitial fluid (ISF), and blood plasma. Since the experiments of Dandy nearly one century ago [3], it is generally agreed that most of the CSF is formed by the choroid plexuses within the cerebral ventricles. More recent reports described that the remaining CSF stems from an extra-choroidal source [4–7]. This extra-choroidal secretion is believed to derive from the ependymal epithelium and possibly the ISF formation across the BBB of cerebral capillaries that subsequently drains into the CSF compartment [8, 9]. However, the exact contribution of extra-choroidal CSF to the total CSF production is still under debate, and may even play a minimal role under physiological circumstances [10].

Recent work by our group showed that the ISF drainage towards the ventricular system is enhanced in the hippocampus of spontaneously hypertensive rats (SHR) [11]. This animal model recapitulates many of the neuropathological characteristics of chronic hypertension as seen in humans with cerebral small vessel disease. Therefore, these animals are frequently used as a model to study the impact of hypertension on the brain [11, 12]. Whether SHR also develop dysfunction at the BBB remains controversial. A greater permeability of the BBB to large molecular weight solutes has been noted in these animals in certain brain areas, such as the deep cortical layer and nuclei in the brain stem, leading to increased penetration of angiotensin II [12, 13]. However, others have shown that the BBB is intact with respect to large solutes when using labelled albumin, and that SHR are actually protected against acute changes in blood pressure [14]. The enhanced ISF drainage towards the CSF compartment in SHR could be another indication for BBB dysfunction, and may result from leakage of water and electrolytes across this barrier [11]. This same study also demonstrated a tendency for a higher brain parenchyma water content in the hypertensive animals. Lastly, tight junction proteins and ion transporters show and altered expression in SHR, associated with augmented oedema formation after arterial occlusion [13].

In the current study, we determined the effects of hypertension on the BBB and blood–CSF barrier (BCSFB) integrity. We hypothesised that increased leakage across the BBB may lead to enhanced ISF formation, resulting in a greater extra-choroidal contribution to the total CSF production. Apart from this, a more leaky BCSFB may in itself lead to an increased CSF production. Both increased ISF and CSF formation could potentially elevate intracranial pressure (ICP). Therefore, we monitored the ICP and estimated the rate of CSF production in SHR and normotensive controls (Wistar Kyoto, WKY). As a second approach, we assessed the penetration of fluorescein, a relatively small molecule, into the brain parenchyma and CSF compartment as an indicator of subtle BBB and BCSFB dysfunction, in the same animals. Since all measurements could be affected by vascular parameters such as the total surface of capillaries, we also examined a number of specific vascular characteristics that may be related to the fluid balance in the brain. Lastly, we determined the ionic composition of the CSF, as this may reflect possible alterations in the ISF electrolyte balance associated with dysfunction of the BBB.

## Methods

### Animals

In this study, a total of 20 rats were used. Male spontaneously hypertensive rats (SHR/NCrl) (n = 10) and Wistar Kyoto rats (WKY/NCrl) (n = 10) were purchased from Charles River Laboratories at 12 weeks of age. Animals were kept until 42 ± 1 weeks old. All rats were housed in groups and were fed standard laboratory food and water ad libitum. The animals were kept at room temperature on a 12-h light, 12-h dark schedule. All experiments were conducted in accordance with the ARRIVE guidelines and European Union guidelines for the care laboratory animals (Directive 2010/63/EU), and were approved by the Academic Medical Center Animal Ethics Committee.

### Chemicals and reagents

Fluorescein sodium salt (Sigma, 0.4 kDa, λ_ex_ 460 nm; λ_em_ 515 nm) was used as a tracer to study blood–brain and blood–cerebrospinal fluid barrier leakage. The dye was dissolved in artificial cerebrospinal fluid (aCSF—135 mM NaCl, 5.4 mM KCl, 1 mM MgCl_2_, 1.8 mM CaCl_2_, 5 mM HEPES, pH 7.4) to a concentration of 40 mg/ml. DyLight^®^594 labelled *Lycopersicon Esculentum* (Tomato) Lectin (Vector Laboratories) was used to label the vascular endothelium.

### Blood pressure and heart rate measurements

Blood pressure and heart rate were measured using a non-invasive tail-cuff system (Kent Scientific) prior to the experiments in conscious rats. As blood pressures in SHR are known to increase from 6 weeks of age and are maintained during adulthood [15], the blood pressure and heart rate was only measured once, prior to the experiments. Animals were acclimated to handling and the restrainer for training period of 4 days prior to the measurements. During the procedure, animals were placed in the plastic restrainer on a heating pad to warm up the tail. Four to 13 BP and HR measurements were recorded and were averaged from each individual rat.

### Surgical procedure

Animals were weighed prior to the experimental procedure. All surgical procedures were performed under isoflurane inhalation anaesthesia (2–3.5%). After induction of general anaesthesia, animals were placed on a heating pad to maintain the core body temperature, which was monitored using a rectal thermometer (Greisinger Electronics). Ophthalmic ointment (Duratears^®^, Alcon) was applied to prevent dryness during the surgical procedure. To study blood–brain and blood–cerebrospinal fluid barrier leakage, fluorescein sodium salt (0.5 kDa) was injected in the dorsal penile vein (40 mg/kg). The animal was turned in the prone position and the head was fixed in a stereotaxic frame (Stoelting). Subsequently, a small longitudinal skin incision was made at the animal’s back of the neck and 10% xylocaine (AstraZeneca B.V.) was applied as additional local anaesthesia. The subcutaneous tissues were removed and the neck muscles were separated in order to reach the cisterna magna. To measure the intracranial pressure (ICP), a 29-gauge stainless steel needle was inserted in the cisterna magna, which was connected to a pressure transducer (Edwards) by stiff polythene tubing. After monitoring the physiological baseline ICP, a second 29-gauge needle was inserted that was connected to a polythene catheter and a U-100 insulin syringe (BD Micro-Fine™). 30 min after intravenous injection of fluorescein, the first cerebrospinal fluid (CSF) sample was collected by gentle aspiration until an ICP of 0.5 mmHg was reached. A third 29-gauge needle connected to a polythene catheter and syringe was inserted into the cisterna magna. A second CSF sample was then collected 60 min after the fluorescein injection. After this, all needles were quickly removed from the cisterna magna and the CSF samples were stored at − 80 °C. The animal was turned into the supine position. DyLight^®^594-labelled *L. esculentum* lectin (1 mg/kg) was injected into the bloodstream via the dorsal penile vein and was allowed to circulate and bind the vascular endothelium for 3 min. Prior to the perfusion fixation, 200 µl of heparin solution (LEO^®^) was injected intravenously to prevent the formation of blood clots. The chest was opened and a blood sample was taken from the vena cava. The blood samples were centrifuged for 3 min at 3000 rpm and the plasma was stored at − 80 °C. Animals were then transcardially perfused with 60 ml heparinised phosphate buffered saline (PBS) and subsequently with 60 ml 4% paraformaldehyde (PFA). The brains were carefully dissected from the skull, weighed, and cut into three coronal pieces using a rat brain slicer matrix (Zivic Instruments). The front part, containing the olfactory bulb and part of the cortex, and cerebellum were snap frozen in liquid nitrogen and stored at − 80 °C until use. The middle part, containing the cortex and hippocampus, was post-fixed overnight in 4% PFA at 4 °C, and subsequently incubated in 30% sucrose for at least 3 days.

### Blood contamination CSF samples

Blood contamination was assessed in all individual CSF samples from both collection time points. The degree of blood contamination in the CSF samples was quantitatively determined by the detection of haemoglobin. 1 µl of the undiluted individual CSF sample was pipetted onto the window of a TrayCell ultra-micro cell (Hellma Analytics), which was subsequently placed in a LAMBDA Bio + spectrophotometer (PerkinElmer). Samples were considered as blood contaminated when the absorbance spectra of haemoglobin were above the detection limit of the spectrophotometer, and were excluded from further studies.

### Ion and protein concentrations in CSF

Undiluted CSF samples were used to determine concentrations of electrolytes, immunoglobulin G (IgG), glucose, and total protein. Sodium, potassium, and chloride concentrations were analysed using an ion-selective electrode, and glucose levels by hexokinase/glucose-6-phosphate dehydrogenase (c702, Cobas^®^ 8000, Roche Diagnostics). Calcium concentrations were measured by spectrophotometry, IgG levels by immunoturbidimetry, and total protein concentrations by turbidimetry (c502, Cobas^®^ 8000, Roche Diagnostics).

### Intracranial pressure and CSF production rate

After insertion of a 29-gauge needle connected to a pressure transducer into the cisterna magna, the ICP was recorded using a PowerLab acquisition system. Chart™ software (ADInstruments) was used to visualise and analyse the data. To measure the baseline ICP, a stable ICP of at least 40 s was selected and averaged. The CSF production rate was determined by calculating the rate of refilling after withdrawal of the first CSF sample. For this, the following equation was used:

$$ {\text{CSF production rate }}\left( {\upmu{\text{l min}}^{ - 1} } \right) = \left( {\frac{{{\text{V}}_{\text{CSF}} }}{{\Delta {\text{ICP}}_{\text{Coll}} }}} \right) \times \left( {\frac{{\Delta {\text{ICP}}_{\text{Refill}} }}{{\Delta {\text{t}}}}} \right) $$

where V_CSF_ is the total volume of collected CSF, ΔICP_Coll_ is the difference in ICP before and after the collection of CSF, and ΔICP_Refill_ is the difference in ICP during a certain period (Δt) in the refill phase.

### Spectrophotometry of plasma, brain and CSF samples

Fluorescence spectrometry was used to quantify the amount of fluorescein in plasma, brain parenchyma and CSF. Plasma and CSF samples were diluted 10,000 and 50 times respectively, in order to be able to measure fluorescence within the range of the spectrophotometer (LS-55, Perkin Elmer). Brain samples comprised the front part of the brain, consisting of the olfactory bulb, cortex and striatum. Care was taken not to include the choroid plexus tissues from the third and lateral ventricle as these tissues may contain high concentrations of fluorescein, thereby interfering with the fluorescein measurements for the brain parenchyma. Brain samples were homogenised in RIPA buffer (150 mM NaCl, 1.0% Triton X100, 0.5% sodium deoxycholate, 0.1% SDS, 50 mM Tris, 1 mM EDTA, pH 8.0) using a Potter–Elvehjem tissue grinder, and an automated homogeniser (Kinematica) to obtain a fully homogenous suspension. Brain homogenates were centrifuged at 7800 rpm for 20 min, the supernatant was removed and used for fluorescence spectrometry.

### Immunohistochemical examination and capillary density quantification

After incubation in 30% sucrose, brains were mounted on a cryostat specimen object disc and subsequently frozen at − 80 °C. Coronal slices of 100 µm were cut on a cryostat (Microm HM 560), transferred to a 48-well plate containing cryoprotectant solution (30% sucrose, 30% ethylene glycol) and stored at − 20 °C until use. Selected sections were collected on SuperFrost glass slides and were allowed to dry for 15 min. Slides were incubated in bisbenzimide (1:100, 3.5 mg/ml, Sigma) for 10 min to visualise the cell nuclei, and coverslipped using fluorescent mounting medium (DAKO). A confocal laser scanning microscope (Leica TCS SP8) was used to acquire brain overview and detailed z-stack images of 70 µm with respectively 10 and 20× objectives. Images were analysed in ImageJ using the automated ‘Blood vessel segmentation and network analysis’ macro developed by S. Tosi.

### Statistical analysis

All data values were reported as mean ± SEM. Data sets were tested for normality by the Shapiro–Wilk test. The group means of parametric data were compared using an unpaired Student’s t-test. The Mann–Whitney U test was applied to test differences between groups in non-parametric data sets. Fluorescein levels were measured in CSF at 30 min and 60 min. Since several samples were contaminated with blood, these data could not be tested with a repeated measurements ANOVA. Therefore, an unpaired Student’s t-test was used. Quantifications of a number of vascular parameters were analysed using repeated measurements ANOVA, followed by Bonferroni’s post hoc tests. Differences between WKY and SHR were considered significant at *p * < 0.05. All statistical analyses were done using GraphPad Prism software (version 7.03).

## Results

### Physical and biochemical parameters

At 42 ± 1 weeks of age, spontaneously hypertensive rats (SHR) had significantly lower brain wet weights than normotensive Wistar Kyoto rats (WKY) (p ≤ 0.01). The body weight did not differ between these strains. Both systolic and diastolic blood, as well as the heart rate, were significantly elevated in SHR as compared to WKY (p ≤ 0.0001) (Table 1).

**Table 1:** Body and brain weights, blood pressure and heart rate in WKY and SHR rats

	WKY (n = 10)	SHR (n = 10)
Weight		
Body (g)	401.0 ± 10.1	398.4 ± 2.6
Brain (g)	2.12 ± 0.03	2.01 ± 0.01**
Blood pressure and heart rate		
Systolic (mmHg)	158.6 ± 2.1	190.5 ± 4.0****
Diastolic (mmHg)	103.9 ± 2.3	145.7 ± 4.5****
Heart rate (bpm)	386.1 ± 8.6	460.9 ± 7.0****

Body weight did not differ between WKY and SHR, while brain wet weights were significantly lower in SHR. Both systolic and diastolic blood pressure, and heart rate were significantly elevated in SHR as compared to WKY. Values are mean ± SEM

*WKY* Wistar Kyoto rat, *SHR* spontaneously hypertensive rat

** p ≤ 0.01 and **** p ≤ 0.0001 vs. WKY (unpaired Student’s t-test or Mann–Whitney U test)

We measured the concentrations of different solutes in CSF samples that were collected during the experiment. CSF glucose levels were remarkably high in both strains, but were significantly lower in SHR when compared to WKY (p ≤ 0.001). In addition, we found a small (< 1%), but statistically significant difference in sodium concentrations between SHR and WKY (p ≤ 0.05), and lower total protein concentrations in SHR (p ≤ 0.05). IgG levels in both strains were below the detection limit of 0.3 g/l. All other solutes were not different between the two groups (Table 2).

**Table 2:** Ionic composition and concentrations of various other solutes in the CSF of WKY and SHR rats

	WKY	SHR
Na^+^	156.3 ± 0.31 (n = 4)	157.5 ± 0.25 (n = 4)*
K^+^	2.76 ± 0.02 (n = 4)	2.77 ± 0.01 (n = 4)
Cl^−^	130.1 ± 0.31 (n = 4)	131.3 ± 0.54 (n = 4)
Ca^2+^	1.29 ± 0.01 (n = 3)	1.27 ± 0.01 (n = 4)
Glucose	8.11 ± 0.30 (n = 4)	5.26 ± 0.20 (n = 4)***
IgG	< 0.30 (n = 3)	< 0.30 (n = 8)
Total protein	0.15 ± 0.002 (n = 5)	0.11 ± 0.01 (n = 7)*

Glucose levels were significantly lower in SHR as compared to WKY, but were remarkably high in both groups

*WKY* Wistar Kyoto rat, *SHR* spontaneously hypertensive rat; IgG, Immunoglobulin G

Na^+^ concentrations were significantly higher in SHR, whereas none of the other ion concentrations were different. Total protein concentrations were significantly lower in SHR. Na^+^, K^+^, Cl^−^, Ca^2+^, and glucose levels are in mmol/L, whereas IgG and total protein are in g/L. Values are mean ± SEM. * p ≤ 0.05, *** p ≤ 0.001 vs. WKY (unpaired Student’s t-test)

### Intracranial pressure and CSF production rate

**Fig. 1:**
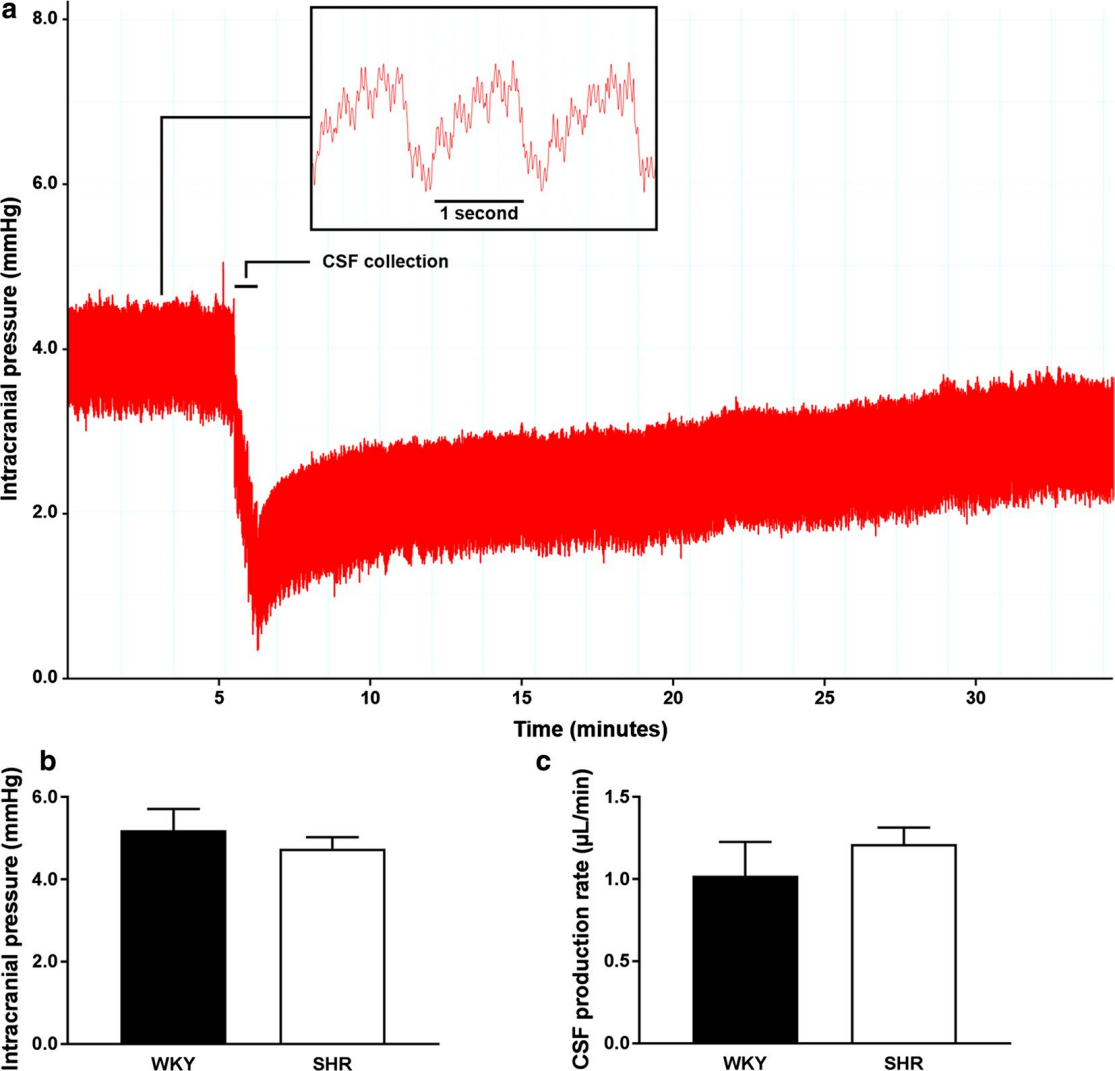
Changes in ICP during collection of CSF from the cisterna magna, baseline ICP, and CSF production rate in WKY and SHR. **a** In this animal, the mean baseline ICP of 4.0 mmHg was measured during the first 5 min of the experiment. Subsequently, a CSF sample was collected over a short period of about 1 min until an ICP of 0.5 mmHg was reached. The animal was then allowed to refill the withdrawn CSF volume for another 30 min, from which the CSF production rate could be calculated. The inset shows a zoom in on the ICP recording, with approximately 1 breath and 4 to 5 heart beats per second. **b** Mean baseline ICP was not different between WKY (n = 10) and SHR (n = 10). **c** Also, CSF production rates did not differ between WKY (n = 6) and SHR (n = 10). Values are mean ± SEM (unpaired Student’s t-test)

Increased fluid leakage from parenchymal and choroidal capillaries as a consequence of hypertension could potentially elevate intracranial pressure (ICP) and CSF formation. Therefore, we recorded the ICP prior to and during withdrawal of CSF from the cisterna magna. Figure 1a shows an example of the ICP recording during the experiment, showing a pulsatile ICP and a clear drop in pressure upon CSF withdrawal. This drop in pressure was followed by a ‘refill-phase’ in which the animal was allowed to restore CSF volume for another 30 min. The inset shows a zoom in on the oscillatory patterns observed in the ICP recordings, which were attributable to the respiration and heart rate of approximately 1 breath and 4 to 5 heart beats per second. Prior to the first CSF collection, we measured the baseline ICP over a stable period of at least 40 s. In the example shown in Fig. 1a, the mean baseline ICP was 4.0 mmHg and was averaged over a period of approximately 5 min. Figure 1b shows the mean baseline ICP in WKY and SHR, with 5.19 ± 0.52 mmHg and 4.75 ± 0.28 mmHg respectively.

The CSF production rate was estimated from the rate of refilling after withdrawal of CSF. Since the CSF is formed by the choroid plexus, together with a possible contribution from ISF formed across the BBB, both an increased BBB and BCSFB permeability could lead to an enhanced CSF formation. However, CSF production rates did not significantly differ between WKY and SHR, with values of 1.02 ± 0.21 µl/min for WKY and 1.21 ± 0.10 µl/min for SHR (Fig. 1c).

### Fluorescein levels as a marker for BBB and BCSFB permeability

**Fig. 2:**
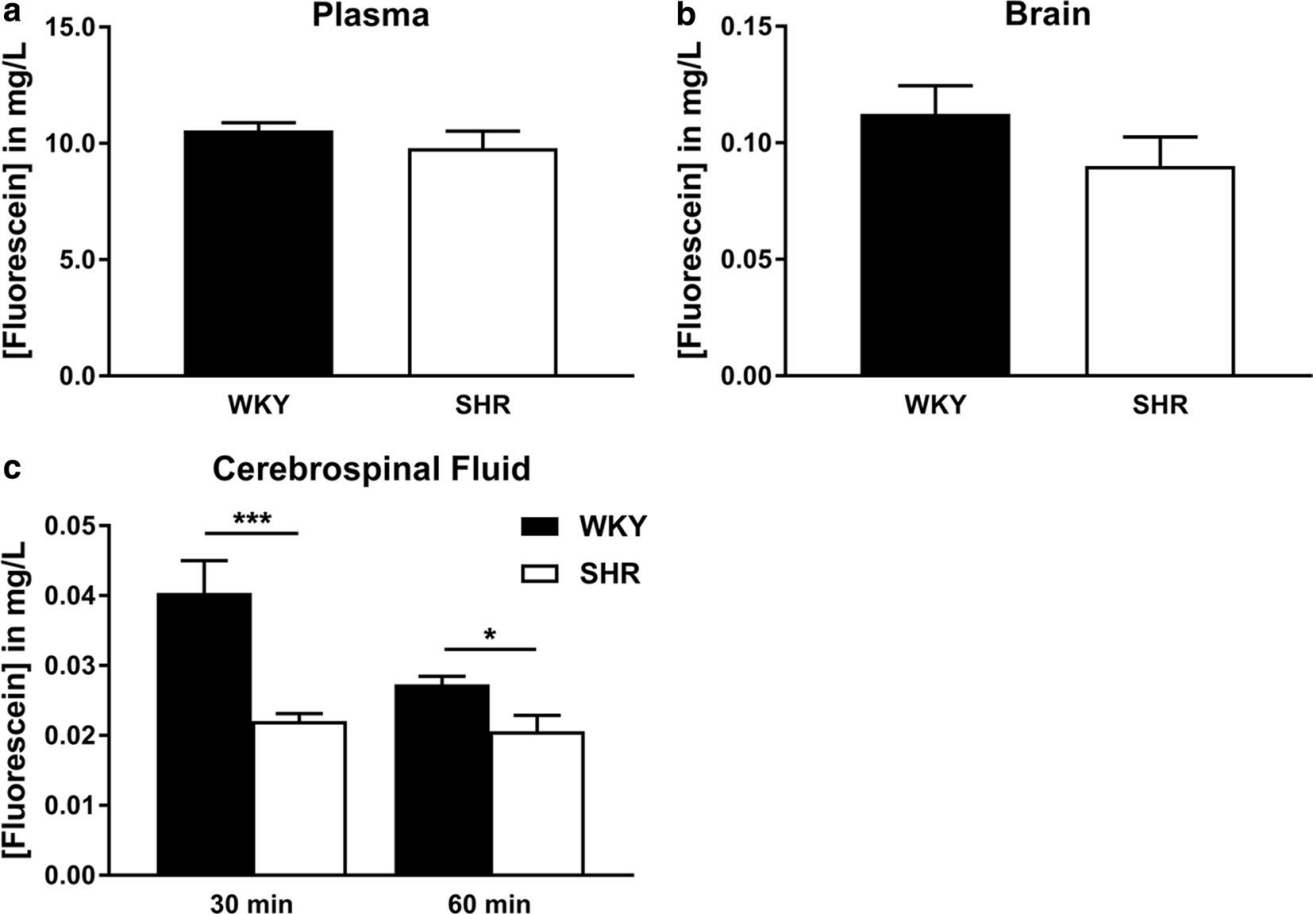
Fluorescein concentrations in plasma, brain and CSF. Fluorescein concentrations were quantified by spectrophotometric analysis in plasma (**a**), brain (**b**), and CSF (**c**) samples of WKY (n = 10 for plasma and brain, n = 7 at CSF 30 min, and n = 5 at CSF 60 min) and SHR (n = 10 for plasma and brain, n = 9 at CSF 30 min, and n = 6 at CSF 60 min). Values are mean ± SEM. *p ≤ 0.05, ***p ≤ 0.001. (unpaired Student’s t-test or Mann–Whitney U test)

BBB and BCSFB permeability to small molecules was assessed by intravenous injection of a relatively small fluorescent tracer (sodium fluorescein, 0.4 kDa) into the bloodstream. The tracer was allowed to circulate for 60 min. After 30 and 60 min, CSF samples were collected from the cisterna magna, while plasma and brain samples were only obtained 60 min after infusion of fluorescein. Spectrophotometric analysis revealed that there were no significant differences in the fluorescein concentrations in both plasma (10.56 ± 0.34 mg/l in WKY vs. 9.80 ± 0.74 mg/l in SHR) and brain (0.11 ± 0.012 mg/l in WKY vs. 0.090 ± 0.013 mg/l in SHR) samples (Fig. 2a and b). In contrast, fluorescein concentration in CSF samples at both collection time points was significantly lower in SHR as compared to WKY. CSF fluorescein concentrations 30 min after fluorescein injection were about twice as high in WKY (0.040 ± 0.0047 mg/l) than SHR (0.022 ± 0.0011 mg/l) (p ≤ 0.001). At the second CSF collection time point, fluorescein concentrations were 0.027 ± 0.0012 in WKY and 0.021 ± 0.0023 mg/l in SHR (p ≤ 0.05) (Fig. 2c).

### Quantification of vascular anatomy

**Fig. 3:**
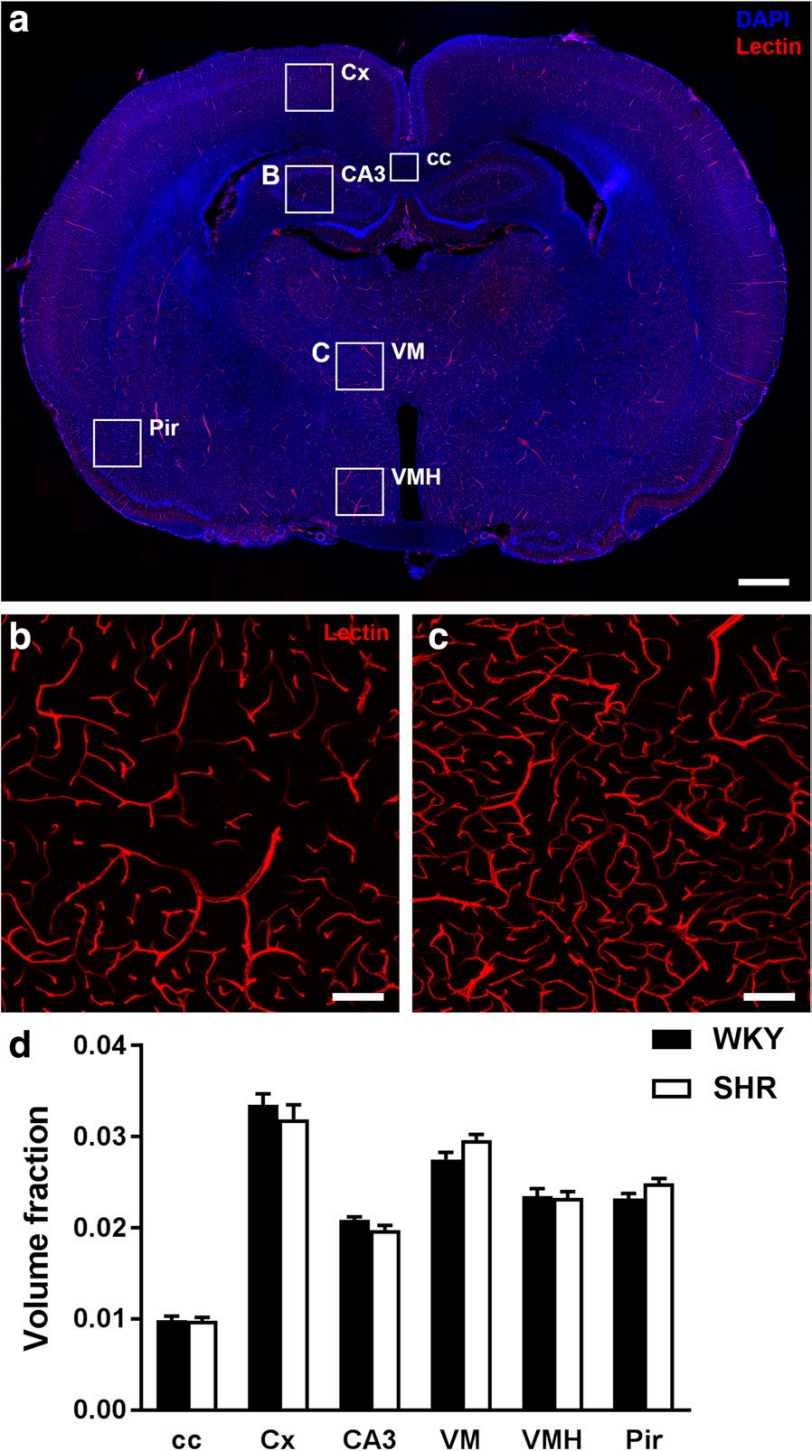
Visualisation and quantification of the rat brain microvasculature. The vascular endothelium was visualised by a DyLight^®^594-labelled *L. esculentum* lectin (red), and cell nuclei by DAPI staining (blue). **a** Overview of a coronal rat brain section indicating the stereotaxic coordinates and 6 different brain regions used to quantify a number of vascular parameters. **b** and **c** Representative images of the lectin staining used for the analysis of vascular parameters in the CA3 and VM respectively. **d** Volume fraction of capillaries did not differ between WKY (n = 10) and SHR (n = 10) in six different brain regions. Values are mean ± SEM (repeated measures ANOVA, Bonferroni’s post hoc tests). cc, corpus callosum; Cx, cerebral cortex; CA3, field CA3 of the hippocampus; VM, ventromedial thalamic nucleus; VMH, ventromedial hypothalamic nucleus; Pir, piriform cortex. Scale bar in **a** represents 1 mm, and 100 µm in **b** and **c**

To further study the impact of hypertension on the cerebral microcirculation, we quantified the mean vessel diameter, number of branches, and volume fraction occupied by vessels. Fluorescently labelled *L. esculentum* lectin was injected into the bloodstream prior to sacrifice and perfusion fixation. This compound rapidly binds the vascular endothelium, which makes it an effective marker of perfused blood vessels. Six different brain regions were imaged to assess whether there were differences in the microvascular density or mean vessel diameter between SHR and WKY. Figure 3a shows an overview of a rat brain section in which these different brain regions are indicated. The images in Fig. 3b and c represent higher magnifications of the lectin staining in the field CA3 of the hippocampus and ventromedial thalamic nucleus respectively. These detail images were subsequently used for the quantification of several vascular parameters in ImageJ. As shown in Fig. 3d, no significant differences between WKY and SHR were found for the vascular volume fraction. Also mean vessel diameter, the number of branches, and total vessel length did not differ between the two strains (Additional file 1).

## Discussion

In the present study, we examined the effects of chronic hypertension on fluid management in the brains of rats at 10 months of age. No differences in either ICP values or CSF production rates were found between SHR and WKY. BBB and BCSFB integrity was subsequently determined by measuring the penetration of sodium fluorescein from the circulation into the brain parenchyma and CSF. Brain fluorescein concentrations in SHR were similar to those found in WKY rats, whereas the levels of this marker were higher in the CSF of the normotensive control animals. Ultimately, we quantified various vascular parameters in different brain regions, which did not reveal any differences between the two strains.

A general characterisation of SHR rats in this study confirmed an elevated systolic and diastolic blood pressure, higher heart rate, and a significant loss of brain mass, when compared to WKY rats. Electrolyte concentrations in the CSF did not reveal substantial differences between SHR and WKY. However, CSF glucose concentrations were significantly lower in SHR. The reason for this difference is unclear, but could hint towards altered metabolic activity between strains. Total CSF protein concentrations were significantly lower in SHR, while IgG levels were below the detection limit in both strains. These observations suggest an intact BBB and BCSFB in SHR, as concentrations of these plasma proteins would expectedly be higher in case of a leaky barrier. Others did find differences in the protein composition of CSF between SHR and WKY, which includes a relatively low level of transthyretin [16]. This could reflect dysfunction of the choroid plexus in SHR, other than increased permeability.

The significantly lower brain weights in SHR suggest cerebral atrophy in these animals. This was also previously reported by Gesztelyi et al. [17] and interpreted as a loss in microvascular tissue and neurons. Consequently, this brain tissue atrophy may result in ventricular enlargement as observed in SHR, a feature that is also observed in hypertensive patients [18–20]. Another possibility is that ventricular enlargement could result from altered fluid production due to hypertension. Thus, hypertension might lead to increased formation of ISF and CSF due to elevated fluid leakage from parenchymal and choroidal blood vessels, which could result in an elevated ICP [20]. To study this, we monitored the ICP via the cisterna magna prior to and during collection of CSF. This showed a highly oscillatory pressure profile generated by the heartbeat and respiration. However, mean ICPs did not differ significantly between SHR and WKY, and correspond well to values reported in the study by Ritter et al. [21]. It may be possible that SHR experience episodic variations in ICP which could not be detected during our experiments.

Determining the CSF production rate is still technically challenging and a number of different techniques have been tested [5], yielding varying production rates. In this study, we estimated CSF production rates from the refill rate after collection of a known volume of CSF according to the procedure described by Masserman [22]. The values we found were similar to those found in rats using the ventriculo-cisternal perfusion method [23, 24], but might be slightly underestimated since the animals were not artificially ventilated. In mice, artificial ventilation was recently found to increase CSF production [25]. The CSF production rates tended to be slightly higher in SHR, but were not statistically different from the normotensive control animals. This suggests that there is no markedly enhanced BBB or BCSFB permeability in SHR, as this would result a more rapid recovery to the ICP after CSF collection, and therefore a higher CSF production rate. However, another possibility may be that the contribution of the extra-choroidal CSF formation across the BBB is too small to detect differences in the presence of a large background of choroidal CSF formation.

We do not expect that differences in ventricular volume affect the calculated CSF production. In the current procedure, we obtained a sample of CSF. From the volume of the sample and the accompanying decrease in pressure we could derive the compliance (delta V/delta P). In case differences in ventricular volume play a role, this is reflected herein. Yet, the compliance was the same in WKY and SHR (data not shown). Thus, refilling of the CSF induces the same gradual increase in pressure in both rat strains, irrespective of the ventricle volume. Another potential limitation is that withdrawal of a CSF sample might lead to a redistribution between ISF and CSF that affects the estimation of CSF production. Thus, the refilling of the CSF compartment after fluid withdrawal might to some extent have originated from the ISF compartment rather than the choroid plexus. Estimation of this contribution would require data on interstitial compliance and resistance for ISF-CSF fluid flow. There are two extreme cases: first, the ISF volume might not change at all in the time course of the experiment, because of low compliance or high resistance. Second, resistance is low enough to allow a rapid equilibrium between these compartments. In either case the presence of the ISF compartment would not affect the estimation of CSF production, although the interpretation of compliance differs. Yet, in intermediate cases of ISF-CSF convection dynamics, a two-compartment model would be needed to more carefully interpret these findings.

BBB and BCSFB integrity was further studied by measuring sodium fluorescein passage across these barriers using spectrophotometry. For the brain tissue samples, care was taken not to include choroid plexus tissue, as preliminary experiments showed that the choroid vascular and stromal tissue contained high concentrations of fluorescein. We used sodium fluorescein as it is described as the most suitable marker to detect more subtle changes in BBB and BCSF integrity because of its low molecular weight of 0.4 kDa. Fluorescein is also less toxic and only weakly binds to plasma proteins such as albumin when compared to Evans blue, which is still the most commonly used dye in BBB permeability studies [26]. One hour after injection of fluorescein, we measured the levels of this dye in plasma, brain parenchyma, and CSF. Measurement of fluorescein in plasma is of importance, as differences in clearance from the blood between SHR and WKY would obscure brain and CSF permeability measurements. However, no differences in plasma fluorescein concentrations were found between SHR and WKY, indicating that the clearance rate from the blood is similar in these strains. Brain fluorescein concentrations were also nearly identical between hypertensive and normotensive rats, and were around 1% of those present in plasma. This finding suggests that the BBB in these rats is still intact, which seems to be in contrast to one study showing decreased BBB permeability [27]. This may relate to a large difference in the time point of the measurements (15 s after tracer infusion versus 30 and 60 min in the current study). Alternatively, the type of anesthesia, the age of the rats, or the use of a different marker for the assessment of BBB integrity, radiolabelled ^14^C-sucrose, which has been criticised [28], may play a role [27]. In a study by Kaiser et al. [12], whole brain permeability to Evans blue was not statistically different between SHR and WKY, whereas the deep cortical region was found to be more permeable in SHR.

Cerebrospinal fluid fluorescein concentrations were significantly lower in SHR as compared to WKY, with roughly a twofold difference at the first collection point. As CSF production rates were similar in the two strains, the latter finding could not be explained by an elevated CSF turnover. While the data might seem to suggest actually a higher leakage of fluorescein in the WKY, a more likely explanation is the increased ventricle volume by about a factor of two in SHR in which the fluorescein dilutes [12, 19, 20, 29]. Taken together, these findings indicate an intact BBB and BCSFB in the hypertensive rats. This is in agreement with a study by Calcinaghi et al. [29], which showed that there is no leakage of the body’s own macromolecules from the circulation across the BBB. Another study by Mueller and Heistad [14] demonstrated an even less permeable BBB in SHR when compared to WKY. The lack of BBB damage might be explained by protective mechanisms, such as inward remodelling of the arteries and autoregulatory responses, which limit increases in pressure at the level of the capillaries.

Chronic hypertension is associated with changes in the structure of the cerebral arteries both in humans and animal models such as the inward remodelling mentioned above [1], but data regarding the capillary network are less clear. A detailed analysis of the microvascular network, including average vessel diameter, number of branches, and volume fraction was done in an automated manner using ImageJ software. This unbiased approach revealed no differences between SHR and WKY in six different brain areas, and is consistent with other studies showing similar findings in the same brain regions [17, 29, 30]. In contrast, Kaiser et al. [12] demonstrated an augmented vessel volume in SHR. This total vessel volume was quantified in the deep cortical region only, which may be one brain area specifically affected by hypertension and might therefore not be considered as a general feature of the hypertensive brain. In addition, the study by Ritz et al. [31] showed higher cerebral small vessel densities in the cortex and putamen of younger SHR, but similar densities in these brain regions in hypertensive and normotensive animals of 9 months of age.

## Conclusions

In summary, we found no evidence for increased BBB and BCSFB permeability to a small compound in hypertensive rats. This was based on quantification of fluorescein leakage from blood into brain parenchyma and CSF. Also indirect consequences of increased fluid leakage, such as elevated ICP or CSF formation, were not evident. However, it remains to be established whether these barriers show differences in permeability to even smaller molecules, such as water, in hypertension. Finally, the brain microvasculature of SHR was not affected in terms of vessel volume, vessel diameter, and the number of branches. From this we conclude that several established features of mature SHR, including the lower brain weight, larger ventricular volume [19, 20], small artery remodelling [12], and increased sensitivity to stroke [32] are not paralleled by a physical loss of BBB and BCSFB integrity.

## Additional file

**Additional file 1: Figure S1.** Number of branches, mean vessel diameter, and total length of cerebral capillaries. Number of branches per tissue volume imaged and mean vessel diameter did not differ between WKY (n=10) and SHR (n=10) in six different brain regions (panels A and B). Panel C shows the total length in SHR as compared to WKY. Values are mean ± SEM (repeated measurements two-way ANOVA, Bonferroni’s *post hoc* tests). cc, corpus callosum; Cx, cerebral cortex; CA3, field CA3 of the hippocampus; VM, ventromedial thalamic nucleus; VMH, ventromedial hypothalamic nucleus; Pir, piriform cortex.

## Abbreviations

BBB: blood–brain barrier
BCSFB: blood–cerebrospinal fluid barrier
CSF: cerebrospinal fluid
ICP: intracranial pressure
IgG: immunoglobulin G
ISF: interstitial fluid
SHR: spontaneously hypertensive rat
WKY: Wistar Kyoto rat
