# Supplementary figures and images for: Blood–brain and blood–cerebrospinal fluid barrier permeability in spontaneously hypertensive rats
Source: Fluids Barriers CNS. 2018 Sep 24;15:26. doi: 10.1186/s12987-018-0112-7 (PMC6151927; doi:10.1186/s12987-018-0112-7)

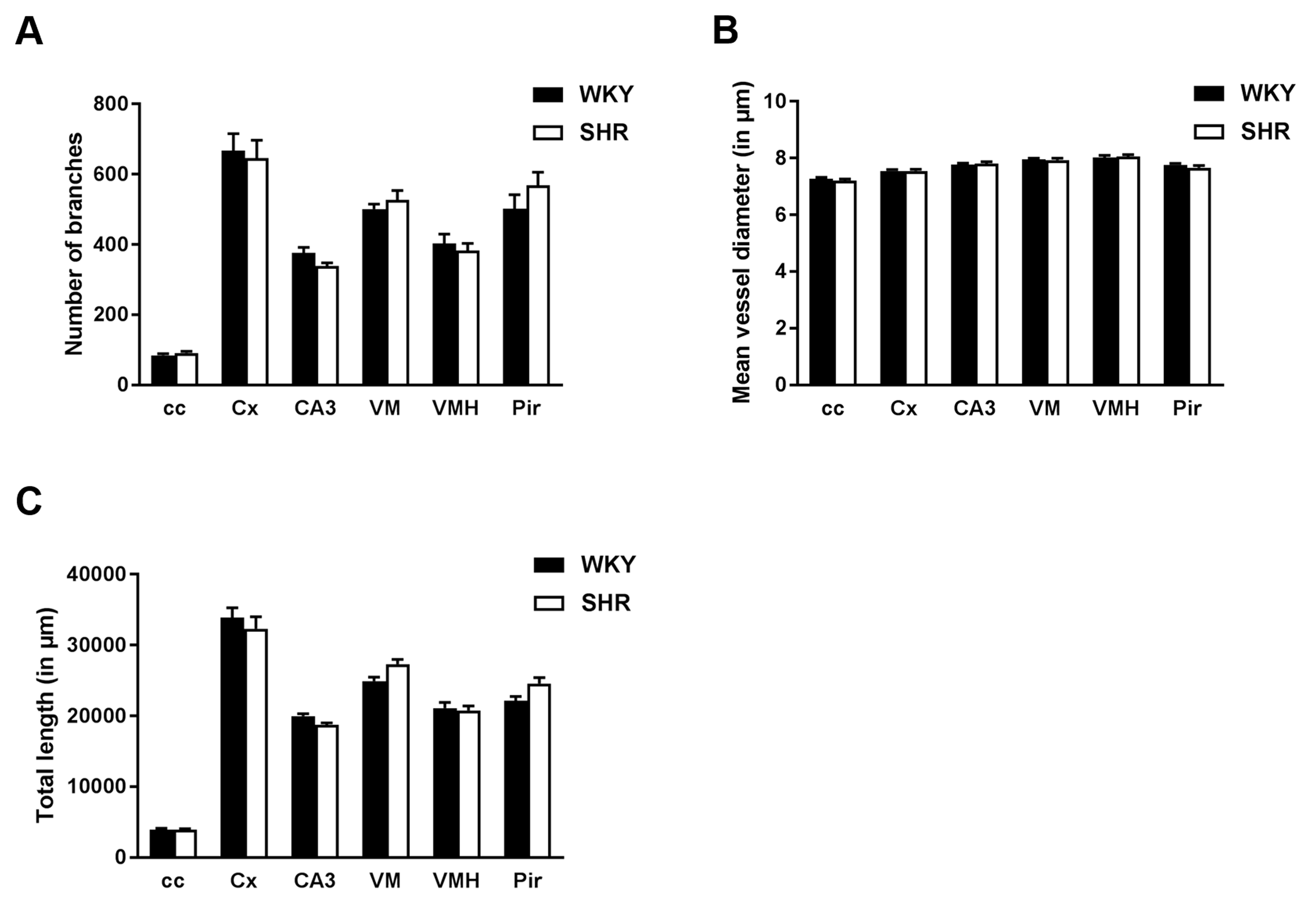

Supplement: Supplementary file 1 — Additional file 1: Figure S1. Number of branches, mean vessel diameter, and total length of cerebral capillaries. Number of branches per tissue volume imaged and mean vessel diameter did not differ between WKY (n=10) and SHR (n=10) in six different brain regions (panels A and B). Panel C shows the total length in SHR as compared to WKY. Values are mean ± SEM (repeated measurements two-way ANOVA, Bonferroni’s post hoc tests). cc, corpus callosum; Cx, cerebral cortex; CA3, field CA3 of the hippocampus; VM, ventromedial thalamic nucleus; VMH, ventromedial hypothalamic nucleus; Pir, piriform cortex. [file 12987_2018_112_MOESM1_ESM.tif]
